# Acceptability and barriers of a GP–physiotherapist partnership in the diagnosis and management of COPD in primary care: A qualitative study

**DOI:** 10.1111/hex.13935

**Published:** 2023-12-08

**Authors:** Lisa Pagano, Zoe McKeough, Sally L. Wootton, Andrew S. L. Chan, Sriram Mahadev, Nicholas Zwar, Deborah Pallavicini, Sarah Dennis

**Affiliations:** ^1^ Sydney School of Health Sciences, Faculty of Medicine and Health University of Sydney Sydney New South Wales Australia; ^2^ Chronic Disease Community Rehabilitation Service Northern Sydney Local Health District Sydney New South Wales Australia; ^3^ Department of Respiratory and Sleep Medicine Royal North Shore Hospital St Leonards New South Wales Australia; ^4^ Northern Clinical School University of Sydney Sydney New South Wales Australia; ^5^ Faculty of Health Sciences and Medicine Bond University Gold Coast Queensland Australia; ^6^ Sydney North Primary Health Network St Leonards New South Wales Australia; ^7^ Ingham Institute for Applied Medical Research Sydney New South Wales Australia; ^8^ South Western Sydney Local Health District Liverpool New South Wales Australia; ^9^ Present address: Australian Institute of Health Innovation, Faculty of Medicine, Health and Human Sciences Macquarie University Sydney NSW Australia

**Keywords:** allied health, COPD, physiotherapy, primary care, qualitative research

## Abstract

**Introduction:**

Chronic obstructive pulmonary disease (COPD) is commonly diagnosed and managed in primary care but there is evidence that this has been suboptimal, with low confidence expressed in providing interventions requiring behaviour change. The aim of this study was to determine the acceptability of a general practitioner (GP)–physiotherapist partnership in the diagnosis and management of COPD in primary care and to explore the experiences of participants during the implementation of the model.

**Methods:**

Semi‐structured interviews were conducted with physiotherapists (*n* = 3), GPs (*n* = 2), practice nurses (PNs) (*n* = 2) and patients (*n* = 12) who had participated in the InNovaTivE Gp‐physiotheRapist pArTnErship for copD (INTEGRATED) trial. We sought to explore participants' views about their experiences and perceived benefits, barriers and facilitators to the implementation of this model of care. Interviews were transcribed, coded and thematically analysed. Synthesis of the data was guided by the Theoretical Domains Framework for clinician interviews and the health belief model for patient interviews.

**Results:**

All clinicians felt that this integrated model helped to optimise care for patients with COPD by facilitating evidence‐based practice. GPs and PNs valued the physiotherapist's knowledge and skills relating to diagnosis and management, which was reported to complement their own management and improve patient outcomes. Patients reported a sense of empowerment following their appointments and acknowledged improved self‐management skills. However, physiotherapists reported many patients were already engaging in positive health behaviours. Responses were mixed on the effectiveness of the model in facilitating teamwork between clinicians with different perspectives concerning management, communication pathways and logistical issues, such as time and room availability, being cited as barriers.

**Conclusions:**

An experienced cardiorespiratory physiotherapist embedded into a small number of primary care practices to work in partnership with GPs for COPD diagnosis and management was acceptable and viewed as beneficial for patients. Barriers relating to logistics and resources remain, which must be addressed to optimise implementation.

**Patient or Public Contribution:**

Patient input was obtained from qualitative feedback from a prior study conducted by two authors and was used to refine the model of care to determine the added value of a physiotherapist integrated into the primary care team. This feedback was also used to refine the interview guides utilised in this study determine the acceptability of this model of care. We had health service involvement from the rehabilitation service of the local health district who were directly involved in determining study aims and establishing the project around the priorities for their chronic disease integration service. For example, this project aimed to engage with a less severe patient population in primary care who would benefit from pulmonary rehabilitation. The findings from this study will be used to further tailor the model of care to the needs of the public and patients. Trial Registration: ACTRN12619001127190

## 1 INTRODUCTION

International and national guidelines exist to guide the diagnosis and management of people with chronic obstructive pulmonary disease (COPD).^1^, ^2^, ^3^, ^4^ However, as the number of COPD cases and the burden on healthcare systems around the world continues to grow, implementation of guideline recommendations remains suboptimal.^5^, ^6^, ^7^, ^8^ The use of spirometry for accurate diagnosis,^9^, ^10^, ^11^, ^12^ initiation of COPD action plans^8^ and referrals to pulmonary rehabilitation (PR)^13^, ^14^ are some examples where evidence‐based COPD management could be improved. Primary care is usually the first point of contact for patients when symptoms occur, and most patients with COPD are managed by their general practitioner (GP). As such, primary care is an ideal setting to implement strategies aimed at optimising diagnosis and management of COPD.

A number of approaches to assist in facilitating best practice of diagnosis and management of COPD have been examined. Interprofessional and team‐based approaches have been suggested as options to improve the management of patients with COPD in primary care and have been recognised by the World Health Organisation as an important component of healthcare with patients who have increasingly complex healthcare needs.^15^ Interviews with healthcare professionals have found that most view interdisciplinary teamwork as positive and generally associate collaboration between different disciplines in the delivery of care with enhanced patient outcomes.^15^ Yet, other studies have found inconsistent implementation of new models of care^16^, ^17^ or little evidence of teamwork to manage COPD.^16^, ^18^

The INTEGRATED (InNovaTivE Gp‐physiotheRapist pArTnErship for copD) study^19^ aimed to examine the effectiveness of a novel intervention in primary care, where physiotherapists work in partnership with GPs to improve identification and management of COPD. Qualitative research that explores the acceptability, challenges and facilitators to implementation of interventions is essential for evaluating the effectiveness of new models of care^20^ and can inform ways in which interventions and service delivery models could be best integrated into clinical practice.^20^ While there are some studies examining the acceptability of case‐finding for COPD,^21^, ^22^, ^23^, ^24^, ^25^, ^26^, ^27^ there is a paucity of literature looking at the acceptability of multidisciplinary models of care in the management of COPD once the diagnosis has been made.^16^ This study is important, as there is currently no published literature on the acceptability of a GP–physiotherapist partnership as well as the acceptability of this model in an Australian primary care setting.

### 1.1 Aims

The aim of this study was to determine the acceptability of a GP–physiotherapist partnership in the diagnosis and management of COPD in primary care. In particular, we wanted to explore the experiences and perspectives of both clinicians and patients during the implementation of the model, identify the extent to which the participating GPs and physiotherapists worked in partnership to diagnose and manage COPD, and identify barriers and facilitators to this model of care.

## 2 MATERIALS AND METHODS

This qualitative study was embedded within a larger pilot study (INTEGRATED trial)^19^ and the detailed methods have been published previously.^19^, ^28^ In brief, four general practices were recruited from a Primary Health Network. Experienced cardiorespiratory physiotherapists were partnered with a GP practice to run a weekly clinic at each participating practice. Adults with a history of smoking and/or a doctor diagnosis of COPD, aged ≥40 years were invited to attend a baseline appointment with the physiotherapist where pre/postbronchodilator spirometry was performed by the physiotherapist.^29^ The intervention for those with COPD confirmed by spirometry involved the physiotherapist and GP working in partnership to develop and implement a plan of care which included the following tailored to patient need: (i) education and advice regarding physical activity (PA) and smoking cessation; (ii) referral to PR; (iii) PA counselling and guided goal setting; (iv) medication review and; (v) the formation or review of a GP management plan or COPD action plan. Patients attended three visits with the physiotherapist, being at baseline, 1 and 3 months.

All participating physiotherapists, GPs, practice nurses (PNs) and patients who completed the 3‐month follow‐up assessment were eligible to participate in a telephone semi‐structured interview and were approached to take part.^30^ Patients were also selected from each general practice to ensure that perspectives could be obtained from each practice location. Clinicians were recruited at the completion of the clinics at their practice and participants were recruited following their 3‐month assessment from June 2019 to July 2020. An interpretive descriptive methodology was employed to understand the different views and experiences of participants.^31^, ^32^ Semi‐structured interviews followed a topic guide (see Supporting Information S1: File 1 and Supporting Information S2: File 2) and were conducted by one researcher with experience in qualitative research (S. D.). Initial topic guides had been designed, piloted and used for data collection in a previous study^18^ by two study authors (S. D. and N. Z.). Interview guides were then modified to incorporate other relevant components based on reviews of relevant literature and theoretical constructs.^33^ Questions were open‐ended with flexibility in the order and wording of questions and probes or additional questions were used to clarify statements where necessary.^34^ Each interview took approximately 15–30 min to complete. Patient interviews were discontinued when the team was satisfied that no new themes had emerged from successive interviews, supporting the conclusion that data saturation had been achieved.^35^ Due to the small sample of clinicians available to take part, a convenience sample of clinicians was used however, data saturation was reached with this cohort.

All interviews were digitally recorded and transcribed verbatim by a professional transcription service. The interviewer made field notes during each interview which included initial thoughts, interpretations and analyses of the data collected. An audit trail of methodological decisions made during research was recorded and reported to assist in increasing the rigour of the study.^36^ This study was conducted according to the Declaration of Helsinki, ethics approval was obtained from the Northern Sydney Local Health District Human Research Ethics committee (HREC/15/HAWKE/434) and the trial was registered with the Australia and New Zealand Clinical Trials Registry (ACTRN12619001127190).

### 2.1 Data analysis

Data were coded using NVivo (QSR International Pty Ltd., [2020] NVivo). The deidentified transcripts were analysed thematically after coding using the method described by Braun and Clarke.^37^, ^38^ The analysis was theoretically underpinned by interpretivism which emphasises that there are varied interpretations which are shaped by different backgrounds and social contexts of each participant.^39^, ^40^ Both healthcare professionals and patient interviews were analysed together to enable data triangulation. Transcripts were read numerous times to ensure immersion before coding. Four transcripts (two clinicians and two patients) were coded by two investigators (L. P. and S. D.) to create the initial coding framework. After discussion and revision of the framework, coding of all subsequent transcripts and creation of categories was then performed by one author (L. P.). Both authors met regularly during this time to discuss analysis, to further develop codes and categories and to ensure all transcripts were coded consistently. Emergent themes were then developed by both authors based on patterns of meaning within the data set and agreed upon consensus by the core research team (L. P., S. D., Z. M. and S. W.). Relevant data was grouped under each theme to ensure an accurate representation of participants' perceptions and experiences.

Synthesis of the data within the health professional interviews was guided by the Theoretical Domains Framework which was developed for use in implementation research to identify influences on health professional behaviour related to the implementation of evidence‐based recommendations.^33^ The health belief model^41^, ^42^ was used to guide coding of the patient interviews and patient demographic data also assisted in understanding any potential patterning around social context.^43^

The practice of reflexivity is an essential component of rigorous qualitative research^44^, ^45^ and disclosure of the researchers' standpoints helps readers to understand how the authors' various viewpoints can shape data interpretation. Therefore, we disclose that four researchers have a background in physiotherapy with experience working with patients with chronic respiratory conditions (L. P., S. D., Z. M. and S. W.). Two researchers are practising respiratory physicians (A. C. and S. M.) and two researchers have a background in primary healthcare as a GP (N. Z.) or through a primary health network (D. P.).

## 3 RESULTS

Nine clinicians (four GPs, two PNs and three physiotherapists) were approached to take part in the qualitative interviews. Two GPs declined to participate due to schedule unavailability and interviews were subsequently conducted with two GPs, two PNs and three physiotherapists from the four general practices. Of the 31 patients selected for the interview, all agreed to participate. A total of 12 patient interviews were conducted, at which point data saturation was achieved. Demographic characteristics of patients are included in Table 1.

**Table 1:** Baseline characteristics of patients that participated in the semi‐structured interviews.

	Total (*n* = 12)
Mean age, years (SD)	74 (10.2)
Sex (% female)	9 (75%)
Place of birth	
Australia	8 (67%)
England	2 (17%)
Scotland	1 (8%)
Canada	1 (8%)
English spoken at home	12 (100%)
Currently married or de facto	5 (42%)
Employment status	
Employed‐full/part‐time/casual	1 (8%)
Retired/pensioner	9 (75%)
Unemployed/student/disability pension/home duties/carer	2 (17%)
Education	
Completed primary school	1 (8%)
Completed high school/some high school	4 (33%)
Tertiary education/vocational training	7 (58%)
Smoking status	
Current	2 (17%)
Former	8 (67%)
Never smoked	2 (17%)

*Note*: Data are presented as number (%) unless indicated otherwise.

Abbreviation: SD, standard deviation.

**Figure 1:**
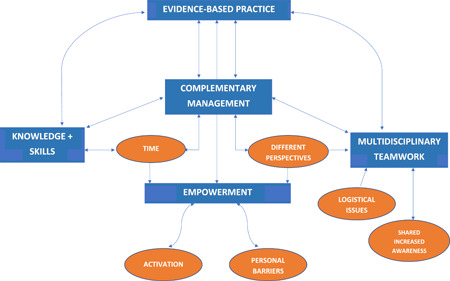
Thematic map of themes and subthemes impacting upon implementation of the integrated model of care.

One overarching theme was identified with four major themes and six subthemes that impacted on implementation and success of this model of care (see Figure 1). The overarching theme was evidence‐based practice (EBP). The major themes identified were: (i) complementary management (subthemes included time and different perspectives of COPD); (ii) knowledge and skills; (iii) multidisciplinary teamwork (subthemes included logistical issues and shared increased awareness); and (iv) empowerment (subthemes included activation and personal barriers). Clinician quotes supporting each theme are presented in Table 2 and patient quotes are presented in Table 3. The corresponding location in the text of each quote is indicated by the label quotation (Q) and the associated number.

**Table 2:** Verbatim quotations from clinicians supporting themes.

Theme	Quotations from physiotherapists	Quotations from GPs/PNs
Overarching theme: *Evidence‐based practice*	Q1: ‘The GP would normally fill in the action plan potentially or start them on a medication […] they were aware that I would refer them on to rehab, or give them physical activity advice, or check their puffer technique, stuff like that’. (PT2)	Q2: ‘Just coming from a different voice and a different perspective than a GP, I think that's really helpful. The team approach to lifestyle management is much more effective than just one voice […] having that breadth of care available to the patients just gives me satisfaction’. (GP2)
Major theme: *Complementing management*	Q4: ‘It was really satisfying to work with particularly the GP practice nurses and the GPs at the practice, because what I found was their knowledge of spirometry and their knowledge of pulmonary rehab and even COPD and quite basic things wasn't quite up to scratch. So, we could step forward and provide them with that education, which was really rewarding, I really enjoyed that, to help develop […] their participation in the management of COPD and referrals to pulmonary rehab’. (PT3)	Q3: ‘It provided an extension of what we do to the patients, I thought that was a very good thing for patients to see and experience, not just coming to a GP and the nurses. So, it was very nice having allied health within the practice […] I think it gives the patient the sense that they're being well looked after. It all fits in with the whole chronic disease team management thing’. (GP2)
		Q5: ‘I think they bring an extra thing that I don't have to the management of COPD. I mean not just from a medical point of view, but the practical application, what a physio can teach people from the point of, how to monitor their lungs, how to improve their lung function, and just improving their general endurance, their exercise endurance. It's a practical skill that I don't have’. (GP1)
Subtheme: *Time*	Q6: ‘I think […] spending that extra time that I think GPs would like to be able to do and also showing all the parts of the COPD guidelines and things, then that's not really being met at the GP practice. So, if that's been done external to that, which it was in the study that we were spending the time going through the individual components, I think that's really beneficial to the patient’. (PT2)	Q7: ‘They just have more time than I have. That's where I found it helpful is that I was quite sure that these guys could teach my patients […] stuff that I didn't have the knowledge for. In a way that would kind of reinforce them and get them involved and get them followed up’. (GP1)
Subtheme: *Different perspectives of COPD management:*	Q9: ‘A lot of them would say, oh yeah, I saw this patient, I noticed your results, we decided not to follow it up, and things like that […] It just becomes a bit frustrating on our end […]. It's only once the patient develops symptoms that affect their quality of life that the GP will actually do something. […] Like, how many times do we see respiratory diseases diagnosed really quite late, because the patients don't develop, or they have symptoms and they ignore them, they're normal? But actually, this could be so much better managed’. (PT3)	
	Q10: ‘I feel like as well how many patients are put on statin and an anti‐hypertensive, on a prophylactic base, and sent to a cardiologist so quickly at the drop of a hat when they are just over 50. But then you have a patient, for example, and we do spirometry and we find obstruction, and we send them back to their GP for a referral to a respiratory physician, and they just don't seem to follow up. But they'll refer them to a cardiologist very easily’. (PT3)	
Major theme: *Knowledge and skills*	Q15: ‘I think we're obviously skilled to do the screening itself, and I guess we should be utilised to try and do that. Obviously take that bit of work load off the medical practitioners where we can. I think from a ‐ I'm a respiratory physio in essence, but I obviously see a lot of lung function, and spirometry results, and so I think we've got a good interpretation of those results too. So, that's a skill that we should be utilising’. (PT1)	Q14: ‘Well, I think their expertise and their knowledge, because I must say a few patients I thought did have COPD, turned out not to, but they did have some impaired lung function. So, it was good to have probably a clearer idea of what their diagnosis was, and what would benefit them really. So, that helped in creating a care plan for the patients as well’. (PN2)
	Q17: ‘I guess, I mean I do that on a day‐to‐day basis really. Especially from a multidisciplinary point of view. Checking inhaler techniques, physical activity advice, exercise advice, action planning type stuff. I guess that's stuff I do all the time. So, I can bring that new knowledge to the table and help managing the patient. Whereas, I think, maybe, the medical point of view is mainly just give them puffers and see you later. So, I think it's just a more holistic approach, as opposed to the medical approach from a GP point of view’. (PT1)	Q16: ‘I think the goals they (patients) had to work toward really helped them improve their general health, as well as their fitness […] Most of the comments were that when they were doing the program […] they did feel a lot better. They had more energy, they were able to do more without feeling breathless’. (PN2)
Major theme: *Multidisciplinary teamwork*	Q23: ‘Because I was comfortable with the doctors there, I'd go and have a chat with them if they were available and just say, look, these are the results, what are your thoughts? […] They were always fine with that’. (PT2)	Q21: ‘(The physiotherapist) would usually talk to whoever it was and get a GP of who the person belonged to, and then we'd sit down and work out a bit of a plan of what to do with this person, how to get them in, how to go through the program with them’. (GP1)
	Q24: ‘What I found was I never got any kind of return formal correspondence from any of the GPs ever, and the only way I could really follow it up would be through the patient when they came for a follow‐up appointment’. (PT3)	Q25: ‘If we had a physio here more often, that we would get to know them, we would talk to them, and we'd be able to have that interaction and conversations, which never happened’. (PN1)
Subtheme: *Logistical issues*	Q28: ‘I wonder if it's actually just a lack of time. I worked in the little room next to [GP] on a Friday and I never saw her stop the entire day’. (PT3)	Q27: ‘I think she was only here one day a week, and our paths didn't cross […] our doctors, I suppose are part time as well, so if she came in on one day, she might never have seen (the GP) […] the communication because of the constricts of our practice and time and space, didn't add to an effective communication’. (PN1)
		Q29: ‘I didn't really want or expect too much interaction regularly with them. I guess they could see that we were pretty busy’. (GP2)
Subtheme: *Shared increased awareness*	Q31: ‘I think how it would change what we do is that we actually need to keep, what I think is a more active role in terms of maintaining lines of communication and keeping them open between us and the GPs in terms of better managing our patients […]. We work very closely with the respiratory physicians, but I think that that same kind of tie needs to be established with the GPs in our health district’. (PT3)	Q30: ‘I'm sending more of the one‐to‐one to the local physio instead of just doing muscular. I'm getting the local physio to be more involved with the chronic lung ones, just really as an educational exercise far more than anything, to check that their techniques are okay’. (GP1)
Major theme: *Empowerment*	Q32: ‘I would say if you have the physio there, then you could incorporate self‐management into it. I feel it's like, when you get that diagnosis from […] a doctor, or if it is a respiratory specialist, it's going to be a bit like, oh yes, and so I want you to go on these three medications […] and I'll see you in six months. Instead of the patient going, oh yeah, I'll take this medication […] start exercising, or refer to pulmonary rehab. So, this patient actually increases their knowledge of their disease, increases their self‐management of their disease, rather than just adhering to their medication and seeing if it works for them’. (PT1)	Q33: ‘I think also, I guess it inspired them a little bit to think that there was room for improvement, you know. Giving them a little bit of hope rather than sort of thinking, well this is my burden more or less and I can't do much else about it’. (PN2)
Subtheme: *Activation*	Q37: ‘They were already very active so we went, ah, probably not going to do much more’. (PT2)	Q39: ‘This is always the downside of doing this type of thing in northern Sydney because smoking rates went down in northern Sydney before they went down in lots of other places […] It's a completely different style of medicine to other parts of Sydney and Australia […] I've certainly seen most of the patients since they've seen the physio so not a lot had to be done with them’. (GP2)

Abbreviations: GP, general practitioner; PN, practice nurse; PT, physiotherapist; Q, quotation.

**Table 3:** Verbatim quotations from patients supporting themes.

Theme	Quotations from patients
Major theme: *Complementary management*	
Subtheme: *Time*	Q8: ‘Just the fact that that (COPD) was concentrated on, which when I go to the GP I don't normally go just for that. That was good that that was really thorough, I was helped in that. I thought it was nice that somebody actually cared about that’. (Pt 4F 81 yo)
Subtheme: *Different perspectives of COPD management*	Q11: ‘And I thought, I'm not really sure I need that (PR) yet […] If I'm exercising and it's flaring up my lungs or something or I can't breathe properly or something, then I'll go back’. (Pt 2F 80 yo)
	Q12: ‘I tend to think what I've got is probably typical of people my age. I think that living in a city with filthy air all your life, and getting to 64, you have to expect to have some impaired lung capacity, as well as just normal deterioration with age. I'm nowhere near needing treatment’. (Pt 5M 63 yo)
	Q13: ‘I felt better, because when you're not well, you don't what's going on. If you go to a place where they know what's going on, it's ‐ it makes you feel a little bit better’. (Pt 6F 50 yo)
Major theme: *Knowledge and skills*	Q18: ‘It was very good from the point of view of – it gave me an insight into what I should be doing’. (Pt 1F 75 yo)
	Q19: ‘I was very keen to start and I gradually increased my walking and some days I actually got up to 6000 steps a day […] I used to walk. I believe in walking and I enjoy it too, but I hadn't for quite a while’. (Pt 2F 80 yo)
	Q20: ‘(The physiotherapist) just gave me a bit more confidence that if I was going to get a cold, I wasn't going to be poorly for months and months and months by following the plan that she put in […], which was amazing’. (Pt 3F 58 yo)
Major theme: *Multidisciplinary teamwork*	Q22: ‘I thought they (GP and PT) […] both seemed to know what was going on, and both were supporting each other really’. (Pt 3F 58 yo)
	Q26: ‘I felt that there probably wasn't enough sort of communication between the physio and the GP’. (Pt 2F 80 yo)
Major theme: *Empowerment*	Q34: Interviewer: ‘It sounds to me since taking part in this, you've been able to self‐manage much more effectively’. Interviewee: ‘Definitely. Because someone's teaching me the things to do, which is good. Instead – because some doctors, they don't know as much as the COPD people’. (Pt 6F 50 yo)
	Q35: ‘Yeah, it's become a habit. I know I have to huff and puff every […] day, and I know when I'm not well or I feel I'm getting a temperature, I have to look after myself that way’. (Pt 8F 87 yo)
	Q36: ‘I have kept it up, which is good […] But actually doing it for that many months, continually, and having this pedometer with me, I think that's made a big change because I've carried it on, and I still do it now. I don't do quite as much, but I still do it regularly now’. (Pt 3F 58 yo)
Subtheme: *Activation*	Q38: ‘I'm fairly certain that I'm fitter than the average 64 year old […] I do a lot of stuff that a lot of 64 year olds couldn't do’. (Pt 5M 63 yo)
	Q40: ‘I knew it before, to be perfectly honest. It was just getting the motivation and the fact that it was available and I took it that it happened’. (Pt 9F 77 yo)
Subtheme: *Perceived barriers*	Q41: ‘Of course it was very bad time for doing a pilot study during the bushfires, I wasn't walking as much as I usually do but yeah […] basically the air was foul […] so it just wasn't a time for increasing one's steps’. (Pt 10M 69 yo)
	Q42: ‘Because of my age and also my debilitating condition, so I've been really isolated […] I'm beginning to find it a bit trying’. (Pt 9F 77 yo)
	Q43: ‘You know, you're busy at work and things, and you just don't have time to go to the doctors, because you're not really dying’. (Pt 3F 58 yo)

Abbreviations: COPD, chronic obstructive pulmonary disease; F, female; GP, general practitioner; M, male; PR, pulmonary rehabilitation; Pt, patient; PT, physiotherapist; Q, quotation; yo, years in age.

### 3.1 EBP

EBP related to the provision of care according to the COPD‐X plan.^1^ All participating clinicians felt that this integrated model helped to optimise care for patients with COPD by facilitating EBP. Clinicians acknowledged that through a shared understanding of each other's roles, they were able to ensure that patients were receiving evidence‐based care (Q1). Clinicians were satisfied with this aspect of the model and valued the joint input from each professional (Q2).

However, while all clinicians acknowledged that the model optimised EBP for patients, the extent to which clinicians were able to work in partnership varied between practices. The major themes presented influenced the perception of EBP, either through promoting or inhibiting EBP in the practice.

### 3.2 Complementary management

Complementary management was identified as a major theme which improved the facilitation of EBP. This theme was closely intertwined with the theme of knowledge and skills as the different clinicians brought various skillsets and approaches to patient care. Both GPs and physiotherapists felt that these different approaches complemented one another where the physiotherapist was able to provide additional management strategies, as well as reinforce strategies previously provided by the GPs and PNs (Q3).

The physiotherapists identified gaps in the current management of COPD, acknowledging areas where their skills could complement those of the GPs and assist in facilitating diagnosis and management according to COPD‐X guidelines^1^ (Q4). GPs valued the physiotherapists expertise in areas of COPD management where they felt they lacked the appropriate skills, which added to patient care. This was particularly evident in the GPs and PNs comments around PA advice and exercise prescription (Q5).

#### 3.2.1 Time

All clinicians commented that a major benefit of this model was the provision of an additional person into the primary care team who had time to dedicate solely to COPD care and this extra time enabled the physiotherapists to successfully complement the GPs' and PNs' management (Q6, Q7). This was also reinforced by patients who valued the time spent on their COPD care (Q8).

#### 3.2.2 Different perspectives of COPD management

The clinicians brought differing perspectives to COPD management which influenced the implementation of EBP. The physiotherapists tended to view patients through a ‘prophylactic’ lens and felt preventative strategies were important to optimise patient outcomes. In contrast, both GPs reported that their patients were ‘fairly normal’ and as such, did not need too much input at that time for their COPD. The physiotherapists felt that this led to lack of follow‐through on management and that the GPs were more likely to act on these recommendations at a later stage, only if something became a problem (Q9). Ultimately, the physiotherapists thought that the management of COPD by GPs was less of a priority than other conditions which they found difficult to understand (Q10).

Many patients also felt that their condition was not severe enough to warrant treatment or that their symptoms did not impact on their daily lives. As such, some could not see the benefits of implementing certain treatments until they deteriorated (Q11). Patients often thought that ageing was the main cause of their symptoms which meant that they regarded their deterioration in symptoms as normal (Q12). In comparison, other patients described worrying symptoms that affected their daily life which they were unsure how to manage. Education provided by the physiotherapists improved their understanding and provided them with tools to manage their symptoms (Q13).

### 3.3 Knowledge and skills

The theme of knowledge and skills was a facilitator of EBP. First, GPs and PNs valued the physiotherapist's skills in accurate diagnosis through the performance and interpretation of spirometry. Both PNs commented on the usefulness of detecting new cases of COPD as this then enabled them to optimise treatment (Q14). The physiotherapists also recognised the value of their knowledge and skills in diagnosis and thought that this could be part of their future role (Q15).

The GPs and PNs acknowledged the usefulness of the physiotherapist's skills in offering a holistic approach and their scope of practice covering multiple aspects of COPD management. They acknowledged that due to the physiotherapist's expertise, there were notable improvements in some patient outcomes (Q16). As this is part of their usual role, the physiotherapists felt this is where they were able to add value to the multidisciplinary team (MDT) (Q17).

Some of the patients interviewed valued how the knowledge and expertise of the physiotherapist helped to guide them in managing their COPD (Q18). This was particularly evident in the education and guided goal setting put in place by the physiotherapists which patients felt helped to increase their overall PA levels (Q19). They also commented on the value of an action plan in managing exacerbations as this made them feel reassured and gave them a sense of control (Q20).

### 3.4 Multidisciplinary teamwork

The theme of multidisciplinary teamwork relates to the dynamics and components that allowed the clinicians in the general practice team to work as an integrated partnership and implement key aspects of EBP. This theme featured prominently in both clinician and patient interviews, however, responses were mixed on the effectiveness of the model in facilitating teamwork between clinicians. Some clinicians reported that the physiotherapists worked well within the practice and felt that the model enabled the development of effective relationships where clinicians could work as a team, enabling the facilitation of EBP (Q21). This idea of effective teamwork was also reinforced by patient responses where interactions between clinicians were perceived as supportive and able to foster a sense of cohesion (Q22).

Opportunistic face to face communication was recognised as an important component in establishing effective working relationships. These opportunistic interactions not only allowed clinicians to discuss patient care, but also helped the physiotherapists to integrate more seamlessly into the primary care team (Q23). However, the opportunity for face‐to‐face communication varied among the practices. For example, in one practice the physiotherapist used the nurse's room when the nurse was not present and so the main method of communication was by written report and both PNs commented that the model would have been more successful if they had been able to spend more time with the physiotherapist. In turn, the physiotherapists expressed that they wanted more interaction with the GP and were disappointed if reciprocal communication pathways were lacking (Q24, Q25).

This was also reflected in some patient responses where they felt that communication between the clinicians needed to be clearer (Q26).

#### 3.4.1 Logistical issues

Logistical issues were reported by all clinicians as the main barrier to establishing effective communication pathways and as such, multidisciplinary teamwork. Room availability within one GP practice was cited as a major issue, where the physiotherapist could only be given space to conduct a clinic for a short period of time per week. In two GP practices, scheduling of clinics was a limitation when working days between staff involved in the study did not always align. Given that the possibility of opportunistic communication and general interactions between clinicians was less in these circumstances, it is unsurprising that staff in these practices felt that the working relationships were less effective, and that the physiotherapist was poorly integrated into the team (Q27).

Both GPs and physiotherapists commented on the ‘busy’ GP caseload which could have been a significant barrier to multidisciplinary teamwork. For example, one GP reported that since they were busy, they were unable to interact with the physiotherapist and did not make attempts at this (Q28, Q29).

#### 3.4.2 Shared increased awareness

In contrast, participating clinicians reported an increased awareness and understanding of each other's roles in diagnosis and management as a result of the model of care. This understanding was viewed to facilitate multidisciplinary teamwork, especially in informing clinicians' future practice where the GPs and PNs said they would be more open to utilising allied health professionals as they better understood their skillset (Q30). Similarly, the physiotherapists reported a greater understanding of how to work better with GPs and the importance of facilitating more effective referral pathways, especially to PR (Q31).

### 3.5 Empowerment

We refer to empowerment as encompassing the processes, motivation and cues to action in which patients take control of decisions regarding their health.^46^ When patients are empowered, they are more likely to adopt certain health behaviours enabling implementation of EBP. All clinicians felt that the physiotherapists instilled a sense of empowerment in patients during appointments. Through education and motivational interviewing, the physiotherapists described the ability to incorporate self‐management components into their treatment and ultimately give patients a sense of ownership over their own care (Q32, Q33).

Crucially, patients acknowledged increased self‐efficacy and improved self‐management skills, valuing the additional education provided by the physiotherapist that was specific to COPD (Q34). Patients described that their appointments with the physiotherapist acted as cues to take action and form habits, particularly with regard to increasing or maintaining PA levels. For many participants, the motivation to sustain these changes was regarded as a major benefit and an understanding of the importance of their own role in their health was apparent (Q35, Q36).

However, patient empowerment was not a decontextualised experience and there were two subthemes identified that either facilitated or inhibited patient empowerment.

#### 3.5.1 Activation

Most patients reported high levels of health activation and knowledge of their disease before participating in the study, with some reporting that they were already engaging in health behaviours important in managing their COPD. Clinicians felt that this directly influenced willingness to change regardless of whether patients were already engaging in positive health behaviours (Q37, Q38). Interestingly, the social context in which participants lived also influenced the views of clinicians and patients of whether change was warranted (Q39). In contrast, some patients reported that despite having prior health knowledge, engaging with the physiotherapists meant that they were able to obtain the motivation to ‘kick‐start’ certain behaviours (Q40).

#### 3.5.2 Perceived barriers

A number of complex, interlinked barriers were identified by participants that negatively influenced empowerment and motivation to adhere to evidence‐based recommendations. Some participants ascribed non‐adherence to extrinsic barriers outside of their control. For example, the study coincided with the increased air pollution during the 2019 bushfires (Q41) and the COVID‐19 pandemic (Q42).

Multimorbidity was reported by most patients where they described their struggle to balance multiple comorbidities with managing their COPD. This was especially evident in the context of PA if participants also had musculoskeletal problems that affected their ability to increase their PA levels. A small number of patients felt that other comorbidities were more severe and required more pressing management than their COPD. Others expressed a lack of fear around their COPD which, combined with other life stressors, prevented them from following‐up on recommendations such as seeing their GP (Q43).

## 4 DISCUSSION

To our knowledge, this is the first study examining the acceptability and barriers to physiotherapists and GPs working in partnership in the management of people with COPD. The findings of this study suggest that this integrated model of GP‐Physiotherapist management was acceptable and able to increase the likelihood of patients with COPD receiving evidence‐based care. This was achieved through clinicians utilising different knowledge and skills in COPD care that complemented one another, and empowering patients through the provision of extra knowledge and motivation. However, differing clinician perspectives of COPD management, communication barriers and logistical issues within the practices were viewed to inhibit EBP.

Our study yielded novel insights about the implementation of an interdisciplinary COPD management model in primary care. A key finding was that the different members of the team ‘complemented’ each other with the different knowledge and skills they each brought to the team. GPs and PNs valued the physiotherapists' skillset, especially the ability to work with patients to encourage behaviour change and increase self‐management behaviours, which is important when considered in the context of previous literature.^13^, ^47^ Qualitative studies examining the implementation of evidence‐based guidelines for COPD reported that some GPs and PNs needed more support and enhanced consultation skills in the areas of management that require complex behaviour change,^47^ such as PA and lifestyle modifications. Further to this, there has been a belief that these interventions are not part of their role or scope of practice.^13^, ^48^ In contrast, the physiotherapists in this study saw these aspects of care as intrinsic to their role which may have been a reason for both patients and clinicians noticing evidence of behaviour change. The fact that the physiotherapists could bring a different skillset to the team to complement primary care management could be of value in future models to support primary care practitioners in these areas of management.

The idea of roles and professional identity can be interlinked with the Theoretical Domains Framework constructs of ‘skills’, ‘knowledge’ and ‘belief about capabilities’. These are core components for members of MDTs as the way in which team members assume their role has been found to influence team effectiveness.^49^, ^50^ Previous literature on MDTs in the areas of social work and nursing have shown that effective MDT members are competent and confident, and this can build trust, respect and collaboration.^49^, ^50^ Yet, a lack of professional confidence can hinder successful implementation of models of care in different ways.^51^, ^52^, ^53^ In our study, the physiotherapists felt that they had the required training in utilising many skills for COPD diagnosis and management and were confident in being able carry out the skills to an appropriate standard. Importantly, this was also noted by the other clinicians which may have resulted in better role clarity and increased trust in the physiotherapists. It is interesting to note that, unlike other studies examining the perceptions of GPs, the GPs in this study did not appear to be as concerned with their knowledge of COPD management according to COPD‐X guidelines^1^ or their ability to provide some aspects of care.^5^, ^6^, ^8^, ^13^ The GPs rather appeared to value the physiotherapists' skillset indelivering advice and complex behavioural interventions advice, especially regarding PA education. This may be a feature of the GPs recruited into the study who had an interest in the area of COPD.

The physiotherapists were able to increase patient self‐efficacy, likely through the capacity to increase patient understanding of perceived benefits and provide cues to action. This theme of empowerment of patients to participate in self‐management interventions such as increasing daily PA levels was not one that resonated with previous MDT research for COPD, but it seemed to reflect a strong link to the health belief model for making sense of individual behaviour. This is a notable finding as despite some evidence of success of brief interventions for PA in primary care,^54^ GPs continue to express low confidence in the provision of specific PA advice and low awareness of certain PA interventions, such as PR.^13^ It is likely since the physiotherapists were highly experienced in cardiorespiratory management and were able to provide individualised advice and exercise prescription for patients, they felt able to motivate patients to take action since there was a specific plan in place and increased accountability. This is unsurprising when examining health behaviours according to the health belief model where individuals are more likely to take action if they believe that a course of action available to them would be beneficial in reducing the severity of the condition and the anticipated benefits outweigh the barriers to uptake.^41^, ^42^, ^55^

The alignment of beliefs and priorities of both patients and clinicians is essential for successful collaboration in the delivery of patient‐centred care. This was highlighted in our study where differences in how practitioners viewed COPD and approached diagnosis and management were perceived by the physiotherapists as a barrier. The GPs and patients in our study focussed mainly on the current problems presented, with less of a focus on lifestyle changes to prevent decline. This is similar to other qualitative literature where varying, less concerned attitudes from some primary care practitioners and patients to aspects of COPD diagnosis and management have been described.^56^ Furthermore, the additional benefit of early diagnosis is seen as insignificant or unlikely to improve outcomes if the patient is currently asymptomatic or only mildly symptomatic.^24^, ^26^

The Theoretical Domains Framework refers to intergroup conflict as a component that may limit transferability of models into adopted practice^33^ so it is important to understand the root of these differences for future models. In our study, rather than these views relating to decreased value placed on preventative care, the impact of ‘environmental context and resources’ was commonly mentioned. Like other studies, time constraints, staffing challenges and reduced resources were cited as barriers to preventative discussions where COPD was often contextualised within a larger picture.^21^, ^24^, ^26^ COPD was viewed as only one condition amongst many others requiring GP attention during short consults, whereas other symptoms the patient may complain of would be viewed as more pressing to address. Placing a physiotherapist who had more dedicated time into the team, meant that they were able to address some of these barriers. However, additional barriers to complete integration were cited which overlap with reasons suggested in other studies, including issues with sustainability. For example, the issue of funding future models was cited by GPs in this study given that allied health professionals are currently poorly integrated into Australian general practice and the current fee‐for‐service model in this context could contribute to inequitable access.^57^ In Australia, PR programmes receive dedicated public funding. However, this funding is only available to hospital‐based programmes, not programmes accessed through primary care. Future models embedded within primary care are likely to need additional funding from other sources such as Medicare initiatives or private health funds to be feasible for both patients attending and clinicians running the services. Interestingly, the issue of future funding was not raised by the physiotherapists in this study, which may be a reflection of their occupation being from a publicly funded service in comparison to Australian general practice.

### 4.1 Limitations

This research focused on one social context where all practices were all located in a relatively affluent area of metropolitan Sydney, with relatively little cultural diversity in our sample of participants. This may limit transferability of findings to other contexts. Due to the small number of clinicians involved in the INTEGRATED study itself, this meant a small pool of GPs, PNs and physiotherapists to interview. The project has, therefore, only touched the surface of the use of physiotherapists in primary care for COPD diagnosis and management. Research that explores these perspectives with a larger sample of clinicians and patients in different sociocultural contexts will result in greater insights into the role of the physiotherapist within different primary care contexts and in turn offer a greater depth to current knowledge.

## 5 CONCLUSIONS

The insights provided from this study revealed that experienced cardiorespiratory physiotherapists embedded into primary care to work in partnership with primary care practitioners in the diagnosis and management of people with COPD was acceptable and viewed as beneficial for patients. The suggested advantages of this model of care stem from the expressed knowledge and skills of the cardiorespiratory physiotherapist enabling participant empowerment, as well as the ability to address some environmental factors such as time and additional personnel, which ultimately led to the facilitation of EBP. As such, this study provides early evidence from a small number of general practices that there is a benefit to adding experienced cardiorespiratory physiotherapists to the MDT in primary care settings. However, in line with previous research, multiple barriers remain, especially in relation to funding models, logistics, communication pathways and resources that must be considered to optimise implementation of interdisciplinary models of care.

## AUTHOR CONTRIBUTIONS

**Lisa Pagano:** Investigation; data curation; formal analysis; writing (original draft preparation). **Zoe Mckeough:** Conceptualization (equal); methodology; funding acquisition; supervision; formal analysis; writing (review & editing). **Sally L. Wootton:** Conceptualistion (equal); methodology; funding acquisition; supervision, formal analysis; writing (review & editing). **Andrew S. L. Chan:** Writing (review & editing). **Sriram Mahadev:** Writing (review & editing). **Nicholas Zwar:** Conceptualization, writing (review & editing). **Deborah Pallavicini:** Writing (review & editing). **Sarah Dennis:** Conceptualization (equal); methodology; funding acquisition; investigation; supervision; formal analysis; writing (review & editing).

## CONFLICT OF INTEREST STATEMENT

The authors declare no conflict of interest.

## ETHICS STATEMENT

This study has been approved by the Northern Sydney Local Health District Human Research Ethics Committee (HREC reference; HREC/15/HAWKE/434) and was conducted in accordance with relevant guidelines and the WMA Declaration of Helsinki. All participants have given their written informed consent to participate in the study. Consent for publication is not applicable.

## Supporting information

Additional File 1: Topic guide for semi‐structured interviews utilised for clinicians,.pdf. Provides the topic guide initially piloted by the research team and used to guide the interviewer in the semi‐structured interviews for clinicians.

Additional file 2: Topic guide for semi‐structured interviews utilised for patients,.pdf. Provides the topic guide initially piloted by the research team and used to guide the interviewer in the semi‐structured interviews for patients.

Additional File 3: Standards for Reporting Qualitative Research (SRQR) checklist,.pdf. Populated checklist of ‘Standards for Reporting Qualitative Research’ reporting guidelines indicating how the manuscript adheres to the relevant guidelines.

## Data Availability

Data will be stored according to and as required by the ethics committee. The data that support the findings of this study are not publicly available due to ethics requirements and are only available from the authors upon reasonable request with permission of the Northern Sydney Local Health District Human Research Ethics Committee.
