# Supplementary material for: Acceptability and barriers of a GP–physiotherapist partnership in the diagnosis and management of COPD in primary care: A qualitative study
Source: Health Expect. 2023 Dec 8;27(1):e13935. doi: 10.1111/hex.13935 (PMC10757211; doi:10.1111/hex.13935)
Supplement: Supplementary file 1 — Additional File 1: Topic guide for semi‐structured interviews utilised for clinicians,.pdf. Provides the topic guide initially piloted by the research team and used to guide the interviewer in the semi‐structured interviews for clinicians. [file HEX-27-e13935-s002.pdf]

# ADDITIONAL FILE 1

## TOPIC GUIDE FOR SEMI STRUCTURED INTERVIEWS UTILISED FOR CLINICIANS

The semi-structured interviews will be conducted as each practice comes to the end of the project; when data collection has been completed for the last patient in the practice. The aims of the interview are to:

- Determine the GP and physiotherapist experience and satisfaction with taking part in the study.
- Explore the effects and value of the physiotherapist input into COPD diagnosis and care.
- Explore the extent of team working for the care of patients with newly diagnosed COPD between GPs and physiotherapists.
- Assess the integrity of the intervention.
- To understand the key barriers and facilitators to partnership management of newly-diagnosed COPD in primary care, from the perspective of both GPs and physiotherapists.

**QUESTIONS:**

***Satisfaction***

Have you found taking part in this study satisfying or unsatisfying? Can you explain why?

What aspects of the study have you found most satisfying?

What aspects of the study have you found least satisfying?

***Team working***

Describe how the process for the diagnosis of COPD worked in your practice.

Do you and the GPs/physiotherapist in your practice work together to make the diagnosis of COPD in patients?

Describe how the management of newly-diagnosed patients with COPD has worked in your practice. Do you and the GPs/physiotherapist in your practice work together to manage patients with newly diagnosed COPD?

Has taking part in the study changed the way you work with the GPs or physiotherapists? If so, in what way has it changed?

***Effects and value of physiotherapy input***

What do you see as the effects and value of physiotherapist input into diagnosis of COPD?

What do you see as the effects and value of physiotherapist input into management of newly diagnosed COPD and existing?

What impact has taking part in the project changed your understanding of physiotherapy / pulmonary rehabilitation / general practice management of COPD?

***Change in practice***

Has taking part in this study had any impact of the way in which you manage COPD in your practice? Can you describe in more detail.

What (if any) are the barriers to managing COPD in your practice?

Now the study has finished in your practice, what are your or your practice's plans for managing COPD in the future

Has taking part in this study had any impact on the way you manage chronic respiratory patients in your practice? Can you describe in more detail.

What about future plans for managing patients with chronic respiratory disease?

Is there anything else you would like to add that has not been covered here?
