# Supplementary material for: Acceptability and barriers of a GP–physiotherapist partnership in the diagnosis and management of COPD in primary care: A qualitative study
Source: Health Expect. 2023 Dec 8;27(1):e13935. doi: 10.1111/hex.13935 (PMC10757211; doi:10.1111/hex.13935)
Supplement: Supplementary file 2 — Additional file 2: Topic guide for semi‐structured interviews utilised for patients,.pdf. Provides the topic guide initially piloted by the research team and used to guide the interviewer in the semi‐structured interviews for patients. [file HEX-27-e13935-s003.pdf]

ADDITIONAL FILE 2

TOPIC GUIDE FOR SEMI-STRUCTURED INTERVIEWS UTILISED FOR PATIENTS

The semi-structured interviews will be conducted as each practice comes to the end of the project; when data collection has been completed for the last patient in the practice. The aims of the interview are to:

- Determine the patient's satisfaction with taking part in the study.
- Explore the effects and value of the physiotherapist input into COPD diagnosis and care.
- Explore the extent of team working for the care of patients with newly diagnosed COPD between GPs and physiotherapists.
- Assess the integrity of the intervention.
- To understand the key barriers and facilitators to partnership management of newly-diagnosed COPD in primary care, from the patient's perspective.

QUESTIONS:

Satisfaction

Have you found taking part in this study satisfying or unsatisfying? Can you explain why?

What aspects of the study have you found most satisfying?

What aspects of the study have you found least satisfying?

Team working

Describe how the process for the diagnosis or management of your COPD worked by the GP and the physiotherapist

Effects and value of physiotherapy input

What do you see as the effects and value of physiotherapist input into your COPD?

What impact has taking part in the project changed your understanding of physiotherapy / pulmonary rehabilitation / physical activity for COPD?

Change in practice

Has taking part in this study had any impact of the way in which you manage your own COPD? Can you describe in more detail.

Now the study has finished in your practice, what are your plans for managing your COPD in the future?

Has taking part in this study changed your understanding of exercise and pulmonary rehabilitation? Can you describe in more detail?

Is there anything else you would like to add that has not been covered here?
